# Where Am I? Searching for the Tangle in the Developmental Topographical Disorientation

**DOI:** 10.3390/neurolint14040067

**Published:** 2022-10-20

**Authors:** Laura Piccardi, Vincenza Cofini, Massimiliano Palmiero, Paola Verde, Maddalena Boccia, Liana Palermo, Cecilia Guariglia, Raffaella Nori

**Affiliations:** 1 Department of Psychology, “Sapienza” University of Rome, 00185 Rome, Italy; 2 IRCCS San Raffaele, 00163 Rome, Italy; 3 Department of Life, Health and Environmental Science, University of L’Aquila, 67100 L’Aquila, Italy; 4 Department of Biotechnological and Applied Clinical Sciences, University of L’Aquila, 67100 L’Aquila, Italy; 5 ItAF Aerospace Test Division, Aerospace Medicine Department, 00040 Pratica di Mare, Italy; 6 Cognitive and Motor Rehabilitation and Neuroimaging Unit, IRCCS Fondazione Santa Lucia, 00179 Rome, Italy; 7 Department of Medical and Surgical Sciences, Magna Graecia University of Catanzaro, 88100 Catanzaro, Italy; 8 Department of Psychology, University of Bologna, 40127 Bologna, Italy

**Keywords:** spatial cognition, topographical disorientation, navigational strategies, DTD, sense of direction, individual differences, poor navigators

## Abstract

The Developmental Topographical Disorientation (DTD) is a pathological condition that impairs an individual’s ability to orient in space, even in the most familiar environments. It is a lifelong selective condition in individuals without brain damage or without impaired general cognitive functions. Here, we aimed at characterizing 54 individuals with DTD identified in a previous study, aged between 18 and 35 years and assessed through a 4-year-long online survey. To this purpose, we compared them with 54 matched healthy participants. We described the demographics, sense of direction, town knowledge, navigational strategies, left-right confusion as well as agnosic disorders (for landmarks, faces and objects). This novel study attempts to characterize the phenotype of DTD, providing an important contribution to the worldwide definition of a condition that was first described only 13 years ago, but which, considering the growing number of cases complaining of the disorder, deserves continuous and increasing attention.

## 1. Introduction

The pathological conditions involving the failure to acquire specific cognitive skills are not new; the first description of dyslexia dates to 1881, although the term was coined six years later by an ophthalmologist from Stuttgart, Germany. Subsequently, specific disorders were described in writing acquisition in both its graphic and orthographic aspects (dysgraphia and dysortographia), in the acquisition of calculations (dyscalculia), and in learning multiplication tables. Finally, beyond learning disabilities in basic literacy skills, pathological conditions were described in the acquisition of abilities such as the recognition of familiar faces (developmental prosopagnosia), music tracks (developmental amusia) and, very recently, also in environmental navigation (developmental topographical disorientation; DTD).

The latter is a pathological condition that impairs the individual’s ability to orient in space. The first case of DTD was described by Iaria and co-workers [1] in a 43-year-old woman (Pt1) who was able to learn and walk a few selected simple routes but was severely disoriented by even minimal deviations from the learned routes. She also showed a severe deficit in the formation of the mental map of a virtual environment; however, she was able to perform similar to controls after an overtraining. Therefore, the authors described her difficulties as a specific impairment in the acquisition of environmental mental representation. Notably, Pt1 showed activity within parietal, temporal and frontal regions during the build-up of the cognitive map, suggesting normal attentional and perceptual processing of the spatial information, but not in the hippocampus and retrosplenial cortex [1]. These latter regions have a crucial role in the formation of environmental mental maps, as shown by neuroimaging studies [2]. Immediately after this first case, Bianchini et al. [3] described the case of a 22-year-old young man (F.G.) with a severe DTD. F.G. showed difficulties in generating the cognitive map of a virtual environment, in drawing the map of his own home, and in describing familiar squares in detail. He was unable to follow a route on a map, to retain previously learned routes, and when he changed houses, he would even get lost inside his new home, especially at night. Differently from Pt1, F.G. performed poorly at three WAIS-R subtests (Picture Completion, Block Design and Object Assembly) and at two mental rotation tasks, all tasks tapping cognitive processes strictly related to spatial navigation. Yet, using an online survey aimed at measuring spatial navigation abilities, Iaria and Barton [4] identified 120 people with DTD. This work demonstrated clearly that the disorder is not isolated but is rather widespread in the population. A very recent study by Piccardi et al. [5] found that DTD is present in 3% of a sample of 1,698 young Italians participants. Interestingly, consistent with the single male cases described in the literature [3,6,7,8,9], Piccardi et al. [5] showed that the DTD is predominant in males.

The cases described so far showed common traits related to the poor or lacking ability to orient themselves in space, all had a difficulty in learning new routes, and all were lost even in familiar environments. For some of the cases, disorientation seemed to depend more on the processing of navigational memory [1,2,6,7], whereas for others on the processing of environmental perceptual features [9], or visuo-spatial features [10]. Barclay et al. [11] suggested the presence of genetic factors in DTD, given that 10 out of 19 relatives of 6 DTD individuals also showed DTD.

Another important characteristic emerging in the literature about single DTD cases is the variability of the severity of the disorder [5], ranging from mild difficulties in acquiring environmental knowledge to the impossibility of learning routes and environments. However, apart from its severity, in all cases, DTD interferes with normal functioning in daily life. In some cases, DTD interferes to such an extent that some individuals with DTD decide to forego working and training opportunities because they are far from home.

Burles and Iaria [12] described a constellation of symptoms in all individuals with DTD. Specifically: (i) losing oneself daily in very familiar surroundings, (ii) their difficulties are present from early childhood, (iii) have no other cognitive deficits, and (iv) have no brain damage or neurological or psychiatric disorders. Furthermore, in 1211 cases of DTD, these authors observed greater levels of neuroticism and negative affect, as well as a poor self-esteem in self-report measures of memory and imagery skills related to objects, faces, and places.

For what attains the neural correlates of DTD, Nemmi et al. [13] found that although the absence of any structural or morphological alterations, performing a task based on navigational sequences activated in an individual with DTD brain areas dedicated to performing action procedures; these areas were not activated in control participants. In this direction, Iaria et al. [14] interpreted the decreased functional connectivity between the hippocampus and the prefrontal cortex as a malfunctioning of areas devoted to navigation and decision-making. Moreover, an rs-fMRI experiment demonstrated aberrant functional connectivity between the medial prefrontal cortex, the posterior cingulate cortex, and the medial parietal and temporal cortices [14].

Considering the importance in everyday life of orienting oneself in the surrounding space and the evidence that in the general population there is a neurodevelopmental disorder that undermines this ability, the present study aims to characterize the 3% of Italian subjects with DTD detected in the previous study by Piccardi et al. [5]. The purpose of this characterization is to highlight commonalities and differences of the individuals affected by this condition with regard to their sense of orientation, their knowledge of the family environment, the way they move in space and their demographic characteristics. Indeed, as also stated by Burles and Iaria [12], only with a well-defined symptomatology scientists and clinicians can develop effective cognitive-behavioural rehabilitation programs. Any attempt to characterize the phenotype of DTD represents, therefore, an important contribution to the worldwide definition of this condition.

## 2. Materials and Methods

### 2.1. Participants

Our sample is constituted of 54 (43 males; 11 females) individuals affected by DTD. Participants were classified with DTD if they reported a total Sense of Direction (SOD) corresponding to 2 Standard Deviations (SD) below the mean (95% CI) at the Familiarity and Spatial Cognitive Style Scale [FCSQ] [15,16]. Furthermore, we took into consideration Iaria and Barton’s [4] following diagnostic criteria: (i) getting lost daily or often (1 to 5 times a week) in the most familiar environments; (ii) problems of spatial orientation must be present from an early age; (iii) no other cognitive difficulties that may affect daily life activities should be present; (iv) absence of conditions affecting the central nervous system, with the exception of migraine. Finally, we also used two following criteria: (v) no psychiatric disorders and psychotropic drug use; (vi) no substance abuse behaviour. The 54 individuals were identified in a previous study [5] in which 1698 Italian participants aged between 18 and 35 years were assessed through an online survey.

A random sample of 54 controls was taken among individuals that did not report DTD in the previous study [5]. Each control was matched to a case of the same age, gender, and instruction level (high/low); therefore, demographic details were not different between groups. Individuals were asked to report if they had suffered from neurological (e.g., head trauma, ischemic attacks, encephalitis, brain infections, pre-perinatal complications) or major psychiatric (e.g., depression, anxiety, psychosis, obsessive-compulsive disorder, eating disorder, post-traumatic stress disorder, schizophrenia, phobias) diseases, traumatic brain injury, history of learning disabilities, alcohol or drug abuses (how often, and which substance, such as cannabis, amphetamines, cocaine etc.). An affirmative answer to any of these questions was a criterion for exclusion from the study. None of the participants reported the problems described above. All of the participants gave their informed consent before their inclusion in the study. The study was performed according to the ethical principles expressed in the Declaration of Helsinki and it was approved by the Department of Psychology, University of Bologna (Bologna, Italy).

### 2.2. Instruments

#### 2.2.1. Self-Reported Assessment of Spatial Orientation Ability

A. The Santa Barbara Sense of Direction Scale (SBSOD) [17].

The SBSOD is a 15-items self-report questionnaire probing spatial and navigational abilities, preferences, and experiences that strongly correlated to the actual navigation ability [18,19]. The score is the average of the responses that ranges between 1 (strongly agree) and 7 (strongly disagree). The higher the score, the more the participants disagree with the statement.

B. The Familiarity and Spatial Cognitive Style Scale (FCSQ) [15,16].

This questionnaire is used to measure different aspects of environmental cognition (sense of direction SOD; town knowledge TK; navigational strategies NS and right-left confusion RLC), such as how people move around the environment, and if they use active (e.g., driving a car; riding a moped; riding a bicycle; riding a motorbike; walking) or passive means of transport (e.g., being a passenger in a car; using a taxi; using a bus; using a train). For each means of transport, participants indicated on a scale from 1 to 5 how often they used it. Based on the frequency of use of the various means, the prevalence of active or passive use of the means of transport is defined. In addition, the questionnaire also measures ‘town knowledge’: participants are asked to think of a town they knew well even if it is different from the one in which they live. See Table 1 for item description.

For each item, participants are asked to respond on a 5-points Likert scale, from 1 to 5: higher scores correspond to higher ability. The Cronbach’s alpha [20] for the total scale is high, ranging between 0.79. and 0.74 [15,16]; the test-retest reliability is also high [16].

#### 2.2.2. Self-Report Assessment of Face Recognition

The structured prosopagnosia scale is a 30-item scale in which participants are asked to indicate on a 5-point Likert scale, ranging from never to always, their face recognition experience as well as object recognition experience, specific strategies used to recognize a person as well as mental imagery skills related to imagine a face [21]. The higher the score on the scale the higher the difficulty.

### 2.3. Procedure

**Figure 1:**
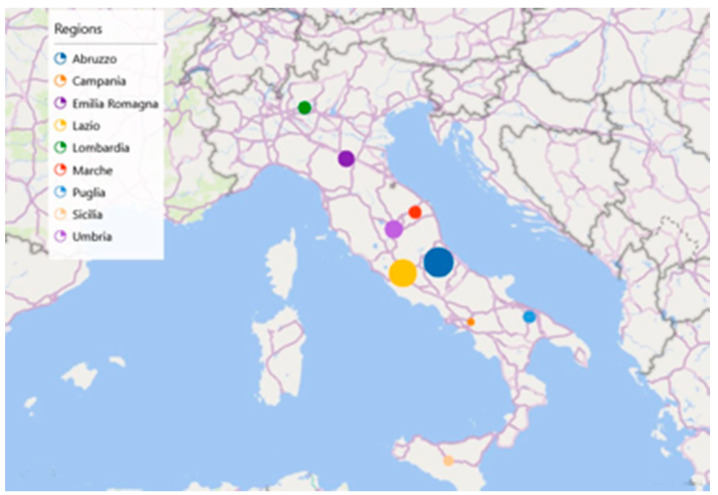
Graphical representation of the distribution of the 54 cases of DTD in Italy.

Participants were recruited between 2016 and 2019 and came from all Italian regions (from North to South, including the Islands) (see Figure 1). Information regarding the survey was basically spread out through social networks, word of mouth and flyers that were distributed in community meeting points, such as local universities, bookshops, cafeterias, public library, and sport clubs. The software Qualtrics (First release: 2005, Provo, Utah, USA, Available at: https://www.qualtrics.com accessed on 23 April 2016)) was used to collect data.

### 2.4. Statistical Analysis

All variables were analysed, and data were reported as means with standard deviations (S.D) or percentages. To compare data between groups, a Two-sample Wilcoxon rank-sum (Mann-Whitney) test and Chi-square or Fisher’s exact test were performed. All analyses were run with Stata 14 Software.

To create the map and the tree map charts, Excel for Microsoft 365 was used.

## 3. Results

Descriptive statistics [means and standard deviations (S.D.)] of all tools are reported in Table 1, Table 2 and Table 3, respectively.

As reported in Table 1, concerning the sense of direction (SOD), DTD participants showed a low ability to use cardinal points (mean = 1.17, S.D. = 0.50) and they had a low SOD (mean = 1.52; S.D. = 0.57), while if they were walking on a well-known route and they found a road closed, they were able to find another way of getting to their destination (mean = 3.26; S.D. = 1.10). With respect to the town knowledge, the neighbourhood knowledge had a mean score (mean = 1.87; S.D. = 0.75) lower than the city (mean = 2.81; S.D. = 0.67), city centre (mean = 2.59; S.D. = 1.07) or the college area (mean = 2.79; S.D. = 1.01) knowledge. Only 3/54 DTD participants (6%) reported that they remember a street if they have gone down the street once and passed there again later; nobody declared to remember it after a year or more. A total of 17 out of 54 DTD participants were able to find their way to the home of a friend that they rarely see; 34 of them reported that the difficulties in this aspect were prevalently related to their street (mean = 1.29; S.D. = 0.67), and neighbourhood (mean = 1.62; S.D. = 0.60). The survey strategy was the navigational strategy less employed (mean = 1.50; S.D. = 0.72), while the participants reported higher scores for route and landmark strategies suggesting a greater use of these navigational strategies in daily life (mean = 3.28; S.D. = 1.07 and mean = 3.15; S.D. = 1.16, respectively).

**Table 1:** Means and S.D. of each item of FCSQ [15,16].

	DTD		No DTD		
Items	N	Means (S.D.) or n (%)	N	Means (S.D.) or n (%)	*p*
	**SENSE OF DIRECTION (SOD)**				
How is your SOD?	54	1.52 (0.57)	54	3.57 (0.98)	<0.001
How is your ability to read a map?	54	1.91 (0.71)	54	3.57 (0.96)	<0.001
Are you able to mentally represent your city? [yes]	54	21 (39%)	54	51 (94%)	<0.001
How is your mental map of the city?	44	1.64 (0.75)	51	3.41 (0.78)	<0.001
How is your ability to give directions?	54	1.93 (0.67)	54	3.39 (0.90)	<0.001
How is your ability to estimate the distance between two places?	54	2.00 (0.70)	54	3.15 (0.90)	<0.001
Do you use cardinal points (N, S, E, W) to orient yourself?	54	1.17 (0.50)	54	1.91 (0.98)	<0.001
How is your ability to find your way back from a familiar place to an unfamiliar one?	53	2.06 (0.63)	54	3.61 (0.74)	<0.001
Are you able to recognize buildings or other landmarks you have only seen rarely?	54	2.30 (0.69)	54	3.70 (0.84)	<0.001
When you are in the interior of a complex building, are you able to tell what there is out in the direction of your gaze?	54	2.07 (0.82)	54	3.44 (0.92)	<0.001
If you are walking on a well-known route and you find a street nearby, are you able to find another way of getting to your destination?	54	3.26 (1.10)	54	2.22 (0.94)	<0.001
	**TOWN KNOWLEDGE (TK)**				
If you have gone down a street once and pass there again at a later time, do you remember it? [yes]	54	3 (6%)	54	47 (87%)	<0.001
the same day [yes]	3	3 (100%)	46	46 (100%)	
some days later [yes]	3	2 (67%)	46	46 (100%)	
some weeks later [yes]	3	1 (33%)	45	26 (58%)	
some months later [yes]	3	1 (33%)	45	13 (29%)	
How well do you know your city?	54	2.81 (0.67)	54	1.94 (0.76)	<0.001
How well do you know the centre of your city?	54	2.59 (1.07)	54	1.61 (0.68)	<0.001
How well do you know the college area (or your job district)?	53	2.79 (1.01)	52	1.85 (0.85)	<0.001
How well do you know your neighbourhood?	54	1.87 (0.75)	54	1.37 (0.59)	<0.001
Would you be able to find your way to the home of a friend you rarely see? [yes]	54	17 (31%)	54	42 (78%)	<0.001
If no, your difficulties concern: your street	34	1.29 (0.67)	12	1.25 (0.62)	0.800
your neighbourhood	34	1.62 (0.60)	12	1.50 (0.90)	0.267
other nearby neighbourhoods	34	3.09 (0.96)	12	2.58 (1.00)	0.121
your city	35	3.91 (0.82)	12	3.50 (0.67)	0.072
Can you explain how to go from your home to the railway station?	54	3.54 (1.27)	54	4.30 (0.92)	0.001
Can you draw a map of this route?	54	2.46 (1.13)	54	3.98 (1.20)	<0.001
Can you explain how to go from your home to the nearest bus stop?	54	2.78 (1.27)	54	4.04 (1.13)	<0.001
Can you draw a map of this route?	54	2.15 (1.20)	54	3.76 (1.36)	<0.001
	**NAVIGATIONAL STRATEGIES**				
How clear are the following directions:					
**ROUTE DESCRIPTION:** “When you leave your hotel, turn right, at the crossroad turn left and go straight ahead for 200 m, then turn left, and after 300 m you will find yourself in Piazza Venezia”.	54	3.28 (1.07)	54	3.89 (0.92)	0.004
**LANDMARK DESCRIPTION:** “When you leave your hotel, go past the coffee shop, and then you will find a book shop. At the first traffic light, look for the bus stop. The square with the big white monument is Piazza Venezia”.	54	3.15 (1.16)	54	3.89 (1.04)	<0.001
**SURVEY DESCRIPTION:** “When you leave your hotel, go west till you reach the pizzeria. From the pizzeria, go east toward the tobacco shop, and you will arrive in Piazza Venezia”.	54	1.50 (0.72)	54	2.39 (1.14)	<0.001
Try to imagine a route you usually take (e.g., home to work, college to cafeteria…):					
**LANDMARK REPRESENTATION:** Do you visualize only the landmarks (e.g., your home, the cafeteria...)?	52	3.77 (0.96)	54	3.54 (1.22)	0.450
**ROUTE REPRESENTATION:** Do you visualize both the landmarks and the route leading to your destination?	52	3.63 (0.86)	54	3.85 (1.02)	0.148
**SURVEY REPRESENTATION:** Do you ever imagine the route as if it were on a map?	53	1.66 (1.02)	54	2.96 (1.30)	<0.001
	**RIGHT-LEFT CONFUSION**				
In everyday life, do you confound right and left?	54	2.11 (1.19)	54	1.89 (1.00)	0.401

**Figure 2:**
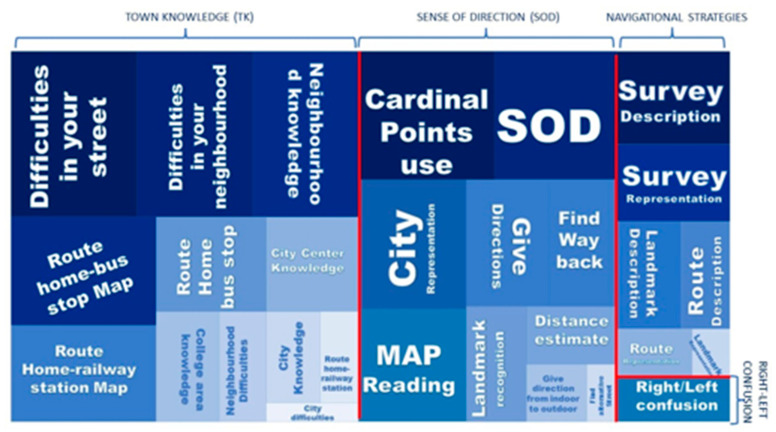
The larger the area and the darker the colour, the greater the difficulty that characterizes DTD in the different aspects of spatial navigation investigated.

Figure 2 shows the representation of symptom severity of DTD according to the different categories of the questionnaire.

The analysis of each item of SBSOD [17] questionnaire showed significant differences between groups for each item. All mean scores in the control group were higher than those of the DTD group.

In the DTD group, the lowest mean scores were for the cardinal points use (mean = 1.46; S.D. = 1.30) and for the item “My ‘sense of direction’ is very good” (mean = 1.69; S.D. = 1.13), indicating a very poor performance in these abilities.

On the contrary, participants showed higher score in “I have a poor memory for where I left things” (mean = 3.22; S.D. = 1.88) and “It’s not important to me to know where I am” (mean = 4.56; S.D. = 1.93), as reported in Table 2.

**Table 2:** Means and S.D. of each item of SBSOD [17].

	DTD	No DTD	*p*
Item	Means (S.D.)	Means (S.D.)	
I am very good at giving directions.	2.09 (1.36)	4.69 (1.82)	<0.001
I have a poor memory for where I left things.	3.22 (1.88)	4.37 (2.06)	0.003
I am very good at judging distances.	2.44 (1.41)	4.50 (1.76)	<0.001
My “sense of direction” is very good.	1.69 (1.13)	4.81 (1.77)	<0.001
I tend to think of my environment in terms of cardinal directions (N, S, E, W).	1.46 (1.30)	2.02 (1.32)	0.002
I very easily get lost in a new city.	2.11 (1.57)	4.76 (1.60)	<0.001
I enjoy reading maps.	2.04 (1.58)	4.50 (1.96)	<0.001
I have trouble understanding directions.	2.91 (1.36)	5.33 (1.37)	<0.001
I am very good at reading maps.	1.93 (1.43)	4.71 (1.73)	<0.001
I don’t remember routes very well while riding as a passenger in a car.	1.89 (1.40)	3.44 (1.86)	<0.001
I don’t enjoy giving directions.	2.07 (1.44)	3.80 (1.91)	<0.001
It’s not important to me to know where I am.	4.56 (1.93)	5.89 (1.48)	<0.001
I usually left someone else do the navigational planning for long trips.	3.15 (2.03)	5.19 (1.86)	<0.001
I can usually remember a new route after I have travelled it only once.	1.94 (0.96)	4.76 (1.64)	<0.001
I don’t have a very good “mental map” of my environment.	2.69 (1.76)	5.37 (1.69)	<0.001

Note: Only 52 participants responded in the Control group.

Table 3 shows that for all items, the mean scores in DTD indicated a poor ability in recognizing faces and objects. Specifically, the greatest difficulties were related to face recognition and concerned the following items: “I have difficulty recognising close relatives from a photo” (mean = 2.41; S.D. = 1.17), “I need excessive time to recognise a person” (mean = 2.28; S.D. = 0.76) and “I have difficulty recognising familiar people I meet out of context” (mean = 1.52; S.D. = 0.64). A total of 13 out of 54 participants (24%) reported to have suffered from these problems since childhood.

**Table 3:** Means and S.D. of each item which investigated the difficulty to recognize faces and objects.

Items	DTD Mean (S.D.) or n (%)	No DTD Mean (S.D.) or n (%)	*p*
I have difficulty recognising from a photo relatives I rarely meet	2.41 (1.17)	1.93 (0.84)	0.034
I happen to be familiar with the faces of people I don’t know	2.85 (0.88)	2.74 (0.93)	0.453
I need excessive time to recognise a person	2.28 (0.76)	1.91 (0.94)	0.012
I happen to mistake strangers for people I know	2.30 (0.92)	1.96 (0.80)	0.058
I have difficulty recognising from a photo friends I rarely meet	1.89 (0.77)	1.67 (0.85)	0.067
If a friend of mine changes his haircut I initially find hard to recognise him	1.43 (0.60)	1.33 (0.73)	0.148
I remember a person’s voice more easily than his/her face	1.93 (0.91)	1.98 (0.86)	0.627
I experience a persistent and irritating feeling of uncertainty in recognising a face that should be familiar to me	2.11 (0.96)	1.87 (1.03)	0.125
In a crowd, I am unable to recognise the face of a known person	2.26 (0.99)	1.83 (0.88)	0.013
In a new/unusual context, I am unable to recognise a known person	2.02 (0.83)	1.78 (0.94)	0.058
I ignore known people because I did not recognise them	2.13 (0.75)	1.93 (1.08)	0.074
I find myself in awkward situations because I cannot recognise a person	2.06 (0.83)	1.87 (0.91)	0.158
If/when it happens to me I adopt special strategies to get out of the embarrassment, e.g., by lying	2.35 (1.15)	1.93 (0.93)	0.054
I consider direct eye contact with my interlocutor important	2.28 (1.25)	1.74 (0.78)	0.039
I use particular strategies to learn to recognise a person, focusing on specific elements of their appearance	2.11 (1.11)	2.44 (1.14)	0.119
I recognise people even in profile	2.57 (0.90)	2.19 (0.75)	0.031
I recognise the emotional state of those in front of me without the need to talk to them	2.61 (0.86)	2.35 (0.68)	0.133
I can tell whether the person in front of me is male or female just by looking at their face (without the help of their voice or other elements)	1.65 (0.70)	1.56 (0.69)	0.383
I have problems recognising particular objects or scenes	2.07 (0.86)	1.61 (0.66)	0.004
I have difficulty recognising close relatives from a photo	1.35 (0.59)	1.24 (0.51)	0.272
I have difficulty recognising familiar people I meet out of context	1.52 (0.64)	1.26 (0.56)	0.013
I am unable to recognise a childhood friend whom I have not seen for several years	2.22 (0.86)	1.93 (0.91)	0.047
I can imagine the face of a familiar person	1.81 (0.87)	1.50 (0.69)	0.039
I could recognise famous actors if I met them in real life	2.67 (0.97)	2.52 (0.93)	0.480
I focus on particular characteristics to recognise a person (by the way he/she walks, voice, haircut, clothing, particular signs such as scars or tattoos)	2.83 (1.16)	3.13 (1.20)	0.208
I can easily recognise my wallet/mobile phone/keyring among many similar objects	1.78 (0.84)	1.59 (0.76)	0.226
I have difficulty remembering the names of friends and family members whom I do not see often	2.33 (1.01)	2.44 (1.06)	0.555
I have problems when I have to recognise my suitcase on the airport conveyor belt	1.87 (0.97)	1.70 (0.86)	0.388
I remember having these problems since I was a child [yes]	13 (24%)	6 (11%)	0.084

Two-sample Wilcoxon rank-sum (Mann-Whitney) test or Chi-Square test.

We used a contingency table to analyse the distribution of the answers to four items which investigate the difficulty to recognize faces and objects, and the ability to recognize buildings or other landmarks seen rarely, evidencing in bold the absolute frequencies of participants who reported “Almost Always” and “Always” problems related to faces or objects recognition and “Never and Rarely” ability to recognize buildings or other landmarks seen rarely (see Table 4). The contingency table showed that 2 out of 54 DTD participants reported that “Almost Always” and “Always”, “I ignore known people because I did not recognise them”. Among them, there was 1 DTD participant with a low ability to recognize buildings or other landmarks seen rarely. Five DTD participants reported problems in recognising objects or scenes and no problems with buildings or other landmarks recognition. Moreover, 44 out of 54 DTD subjects could easily recognise their wallet/mobile phone/key ring among many similar objects and 31 out of 54 have difficulty to recognize buildings or other landmarks they see rarely. The number of participants who had problems recognising their suitcase on the airport conveyor belt was 3 out of 54 DTD and only 2 of them also have difficulty in recognizing buildings or other landmarks seen rarely.

## 4. Discussion

Orienting oneself well in space requires a series of cognitive processes (i.e., recognizing routes and landmarks, identifying the correct direction as well as the final destination, updating the environmental properties and one’s position in the environment) [22] that individuals with a good SOD perform spontaneously without realizing the great complexity of this human skill.

The naturalness by which spatial orientation takes place is, however, not experienced by all people. In fact, there are individuals with a poor SOD and others suffering from a lifelong inability to orient; specifically, these individual show DTD.

In this report, we analyse demographic features and self-report measures of SOD, town knowledge and navigational strategies as well as other related disorders (i.e., inability to recognize faces and objects and left-right confusion) in a sample of young Italians suffering from DTD. In addition, this sample with DTD was compared with a sample matched for age, gender, and education level without DTD to better understand how significantly individuals with DTD differ from individuals without this disorder.

Differently from Burles and Iaria [12], we found that males are more affected than females by DTD with a ratio of 4:1. This finding is in line with the single cases described in literature, given that we identified more males [3,6,7,9] than females [1,8,23]. It is worth emphasizing that the two samples are not equivalent. Burles and Iaria [12] collected 1211 individuals with DTD over 10 years by including individuals up to 65 years old. On the contrary, here, we collected 54 individuals with DTD over 4 years by including individuals up to 35 years old. We decided to narrow down the age range because with a remote self-assessment it is not possible to exclude poor navigators with an onset of cognitive decline, being unable to carry out an in-depth neuropsychological testing and a clinical interview, but collecting just an initial self-reported anamnesis. Furthermore, we also decided not to include individuals under the age of 18 in the study for legal reasons (that would require both parents’ consent to study). If we had included individuals with the same age range as in Burles and Iaria’s study, we would probably have a larger number of individuals proportional to their study conducted over 10 years (i.e., 4:10 = x:1211; x = 484). Furthermore, looking at the 2019 ISTAT (https://www.tuttitalia.it/statistiche/popolazione-eta-sesso-stato-civile-2019/ accessed on 8 October 2022) data for the Italian population, there is a prevalence of Italian males up to 39 years of age, with a range of 50.2% up to 52.4%, which could partially explain the higher number of males trend that reverses from 40 years of age onwards.

Concerning SOD, we found that individuals with DTD estimate themselves very poor in spatial orienting and in using cardinal points. In fact, individuals with DTD differ significantly in all self-rated aspects of SOD on both scales compared with the control group. This result differs from the review by Hegarty and Waller [24] in which they reported very low (r = 0.30) and usually non-significant correlations between small-scale spatial ability and environmental spatial ability. Moreover, other studies weakly demonstrated prediction between spatial ability tests and real-world navigation [25,26,27,28]. Individuals with DTD would also appear to differ in these aspects of visuospatial cognition, so their impairment would include not only large-scale purely environmental aspects but also small-scale visuospatial abilities; however, these do not always emerge from individual case studies.

With respect to TK, individuals with DTD report to be more compromised in navigating their streets and neighbourhoods and declare a better general knowledge of their whole cities. This is in line with the fact that they state that they get lost in familiar environments and that they hardly ever go outside their environments. Therefore, we could imagine that they have a semantic rather than spatial knowledge of the city, differently from that of their neighbourhoods. Moreover, they certainly experience a failure more often in the familiar surroundings than in the city in general where they venture out more rarely or travel in the company of others. Since they experience a failure more often in familiar environments, they indicate their deficit as more severe in these environments with respect to others. However, a comparison with the group of individuals without DTD shows that individuals with DTD differ significantly in their knowledge of the city in all its aspects, thus highlighting that although they feel more distressed in the most familiar environments when compared with a control group, their self-assessment is significantly worse everywhere. Likely, their feeling is related to the fact that in well-known environments their awareness of their difficulty increases.

Regarding navigational strategies, DTD report greater difficulty with survey strategies that are also those used by the most skilled navigators such as military pilots and engineers (see [29,30]). The non-use of survey strategies is also in line with their reported failure to use cardinal points, which are used with great ease by good navigators. In this respect, our sample is similar to that of Burles and Iaria [12], who had difficulty reproducing the explored virtual place map, which is also a survey skill. This is also confirmed by the fact that in their use of the stated navigational strategies they differ significantly from the control group only in survey-type strategies.

Exploring the difficulty in recognizing landmarks, faces and objects, we found a number of individuals that complain of difficulties in recognizing landmarks and faces more than objects. However, these difficulties are not significant and are present only in a small number of individuals suffering from DTD. Nevertheless, a comparison with the control group shows a different situation because participants with DTD are different from the control group in their ability to recognize relatives rarely seen, childhood friends, or familiar people met out of the context. They have difficulty in recognizing scenes and objects too. They do not find useful to look at a person in the eye, which is the best strategy for recognizing the other person, and consequently by this comparison they would also seem to have problems in recognizing faces and objects. An interesting finding also emerges with respect to the significant difference in the ability to mentally imagine faces. This has an important practical implication, and it is in line with a recent study describing the effectiveness of imaginal training in the rehabilitation of acquired topographical orientation disorder [31]. From these findings, it would seem that such training could also be effective in reducing the symptoms of DTD. In addition, even the case described by Piccardi et al. [9] that had both developmental landmark agnosia and developmental prosopagnosia, the treatment of developmental prosopagnosia had produced positive effects on developmental landmark agnosia by greatly reducing its symptoms. However, with respect to single case studies, just two cases showed the co-occurrence of a landmark agnosia and prosopagnosia [9,23]. This co-occurrence is also reported in acquired deficits [24,25] indicating a general difficulty in accessing similar stored representations.

Certainly, there are different subtypes of DTD as already hypothesized by Piccardi et al. [9], suggesting the existence of a true taxonomy of DTD. Probably the subtype with agnosia for landmarks and prosopagnosia is less common than that with a spatial orientation disorder due to a poor environmental representation. The distinction between disorders involving memory and recognition of the environment is not new in clinical neuropsychology; in fact, Farrell [32], discussing acquired topographical disorientation, distinguished between two main types of disorders: a landmark amnesia, in which patients recognize places but are unable to point the direction to follow for reaching two following landmarks, and a landmark agnosia in which patients are unable to recognize the place even if they were able to represent the spatial arrangements of landmarks and the relation between target and landmarks. It is also true that the instruments used to assess the presence of DTD in our report are thought to provide a general screening and not to characterize different subtypes of DTD, an aspect on which future research should focus.

Left-right confusion, on the other hand, is little reported by individuals with DTD and this is also confirmed by the absence of significant differences with the control group.

Individuals with DTD live with a condition that is difficult to share with others because it is little understood, even in the medical field. Yet in its more severe form, this condition is particularly disabling because it prevents individuals from making choices that could lead them to environments far from the familiar ones, thus limiting their autonomy, professional success and possibility of meeting the partner that suits them best. Even a banal outing with friends can become tiring; some DTD report that in order not to become lost, they even avoid going to the bathroom for hours if they are in unfamiliar places. Others report that they find it distressing to confess their condition to recently met friends because they fear being judged as incompetent. Individuals with DTD, as individuals suffering from other developmental specific disorders, reported the use of compensatory strategies, but very often they are situational strategies or verbal strategies and are not sufficient to improve their spatial navigation [3,10,12]. On the other hand, other neurodevelopmental disorders have also taken time to be accepted by society and receive the right support. Just thinking of dyslexia, which until recently was not understood by the school system.

DTD also deserves to be known by the general population to reach out to all the undeclared cases and to provide them with interventions aimed at reducing the discomfort of this condition and allowing to conduct a normal life.

## 5. Conclusions

In the present report, we characterize demographic features, SOD, TK, navigational strategies and left-right confusion as well as agnosic disorders in young Italian individuals suffering from DTD compared to a control group matched for age, gender and educational level.

Our findings show that DTD, in our sample, is more diffuse in male than female participants. They have a greater difficulty orienting themselves in familiar environments and are unable to use cardinal points, thus showing a greater impairment of the allocentric reference system. In fact, environmental objects may be located according to two different frames of reference, namely allocentric (world-centred) and egocentric (body-centred) frames of reference [33,34,35]. Generally speaking, individuals with DTD attempt to use egocentric frame of reference, that is to say route strategies, even if they also often fail in using them. Some subtypes of DTD, such as developmental landmark agnosia, seem to be less diffuse and rarer. 

Although this study is relevant because it adds information about a disorder that was described only a decade ago, it is not without limits. First of all, we obtained individual self-reports and not performances based on behavioural tasks. Indeed, although the SOD is mainly measured by asking participants to self-assess their SOD, adding behavioural tasks would have allowed us to take into account clusters of deficits characterizing DTD subtypes. On a positive note, we used the SBSOD [17] and FCSQ [15,16], which have a good internal consistency and test-retest reliability and correlate with several navigational tasks (e.g., to identify one’s position in the environment; to draw maps etc.). Another limitation may be the time span of only 4 years compared to the 10 years of Burles and Iaria’s observation [12]. Nevertheless, the pandemic situation undermined the possibility of having a sample comparable to the previous one, because the COVID-19 virus increased the number of individuals suffering from spatial anxiety [36] due to the government-imposed restrictions that required locked-down. Therefore, we decided to stop to collect data during the COVID-19 pandemic. With respect to the small number of subjects with DTD, it reflects the numerosity of other neurodevelopmental disorders, such as that of developmental prosopagnosia (2–2.9% of the population [37,38]). In the future it will be appropriate to contact all individuals with DTD and ask them to complete a more comprehensive navigational battery that would allow for a better diversification of the disorder itself.

Undoubtedly, further studies should be conducted on DTD both to investigate its nature and to propose rehabilitation and remote support tools to help individuals so that they are not left alone with their condition.

## Figures and Tables

**Table 4:** Contingency table. Each cell indicates the number of participants with DTD who answered in the respective combination of the two questions.

		FCSQ Item				
Prosopagnosia Items		Are You Able to Recognize Buildings or Other Landmarks You Have Only Seen Rarely?				
I ignore known people because I did not recognise them		Never	Rarely	Sometimes	Almost Always	Always
	Never	2	5	2	1	0
	Rarely	1	19	7	2	0
	Sometimes	1	8	4	0	0
	Almost Always	0	1	1	0	0
	Always	0	0	0	0	0
I have problems recognising particular objects or scenes						
	Never	2	9	2	0	0
	Rarely	1	15	11	2	0
	Sometimes	0	5	1	1	0
	Almost Always	1	4	0	0	0
	Always	0	0	0	0	0
I can easily recognise my wallet/mobile phone/keyring among many similar objects						
	Never	0	0	0	0	0
	Rarely	1	0	1	0	0
	Sometimes	0	5	2	1	0
	Almost Always	0	12	8	0	0
	Always	3	16	3	2	0
I have problems when I have to recognise my suitcase on the airport conveyor belt						
	Never	1	16	5	2	0
	Rarely	1	9	7	0	0
	Sometimes	1	7	1	1	0
	Almost Always	1	1	0	0	0
	Always	0	0	1	0	0

## Data Availability

Data are available requiring them to the corresponding authors.

## References

[1] Iaria G., Bogod N., Fox C.J., Barton J.J. (2009). Developmental topographic disorientation: Case one. Neuropsychologia.

[2] Iaria G., Chen J.K., Guariglia C., Ptito A., Petrides M. (2007). Retrosplenial and hippocampal brain regions in human navigation: Complementary functional contributions to the formation and use of cognitive maps. Eur. J. Neurosci..

[3] Bianchini F., Incoccia C., Palermo L., Piccardi L., Zompanti L., Sabatini U., Peran P., Guariglia C. (2010). Developmental topographical disorientation in a healthy subject. Neuropsychologia.

[4] Iaria G., Barton J.J. (2010). Developmental topographical disorientation: A newly discovered cognitive disorder. Exp. Brain Res..

[5] Piccardi L., Palmiero M., Cofini V., Verde P., Boccia M., Palermo L., Guariglia C., Nori R. (2022). “Where am I?” A snapshot of the Developmental Topographical Disorientation among young Italian adults. PLoS ONE.

[6] Bianchini F., Palermo L., Piccardi L., Incoccia C., Nemmi F., Sabatini U., Guariglia C. (2014). Where am I? A new case of developmental topographical disorientation. J. Neuropsychol..

[7] Palermo L., Piccardi L., Bianchini F., Nemmi F., Giorgio V., Incoccia C., Sabatini U., Guariglia C. (2014). Looking for the compass in a case of developmental topographical disorientation: A behavioral and neuroimaging study. J. Clin. Exp. Neuropsychol..

[8] Conson M., Bianchini F., Quarantelli M., Boccia M., Salzano S., Di Vita A., Guariglia C. (2018). Selective map-following navigation deficit: A new case of developmental topographical disorientation. J. Clin. Exp. Neuropsychol..

[9] Piccardi L., De Luca M., Di Vita A., Palermo L., Tanzilli A., Dacquino C., Pizzamiglio M.R. (2019). Evidence of taxonomy for Developmental Topographical Disorientation: Developmental Landmark Agnosia Case 1. Appl. Neuropsychol. Child.

[10] Iaria G., Incoccia C., Piccardi L., Nico D., Sabatini U., Guariglia C. (2015). Lack of orientation due to a congenital brain malformation: A Case Study. Neurocase.

[11] Barclay S.F., Burles F., Potocki K., Rancourt K.M., Nicolson M.L., Bech-Hansen N.T., Iaria G. (2016). Familial aggregation in developmental topographical disorientation (DTD). Cogn. Neuropsychol..

[12] Burles F., Iaria G. (2020). Behavioural and cognitive mechanisms of Developmental Topographical Disorientation. Sci. Rep..

[13] Nemmi F., Bianchini F., Piras F., Péran P., Palermo L., Piccardi L., Sabatini U., Guariglia C. (2015). Finding my own way: An fMRI single case study of a subject with developmental topographical disorientation. Neurocase.

[14] Iaria G., Arnold A.E., Burles F., Liu I., Slone E., Barclay S., Bech-Hansen T.N., Levy R.M. (2014). Developmental Topographical Disorientation and decreased hippocampal functional connectivity. Hippocampus.

[15] Piccardi L., Risetti M., Nori R. (2011). How well do you know your city? Effects of familiarity on environmental representations: A self-report study. Psychol. Rep..

[16] Nori R., Piccardi L. (2012). Il senso dell’orientamento: Quanto conta la familiarità con l’ambiente?. GIP.

[17] Hegarty M., Richardson A.E., Montello D.R., Lovelace K., Subbiah I. (2002). Development of a self-report measure of environmental spatial ability. Intelligence.

[18] Janzen G., Jansen C., van Turennout M. (2008). Memory consolidation of landmarks in good navigators. Hippocampus.

[19] Wegman J., Fonteijn H.M., van Ekert J., Tyborowska A., Jansen C., Janzen G. (2014). Gray and white matter correlates of navigational ability in humans. Hum. Brain Mapp..

[20] Nunnally J.C., Bernstein I.H. (1994). Psychometric Theory.

[21] Piccardi L., Palermo L., Palmiero M., Guariglia P., Nori R. Normative data of “The structured prosopagnosia scale”.

[22] Golledge R.G. (1999). Wayfinding Behavior: Cognitive Mapping and Other Spatial Processes.

[23] Rusconi M.L., Fusi G., Stampatori C., Suardi A., Pinardi C., Ambrosi C., Costa T., Mattioli F. (2021). Developmental Topographical Disorientation with concurrent face recognition deficit: A case report. Front. Psychiatry.

[24] Hegarty M., Waller D.A., Shah P., Miyake A. (2005). Individual Differences in Spatial Abilities. The Cambridge Handbook of Visuospatial Thinking.

[25] Hegarty M., Montello D.R., Richardson A.E., Ishikawa T., Lovelace K. (2006). Spatial abilities at different scales: Individual differences in aptitude-test performance and spatial-layout learning. Intelligence.

[26] Kozhevnikov M., Motes M.A., Rasch B., Blajenkova O. (2006). Perspective-taking vs. mental rotation transformations and how they predict spatial navigation performance. Off. J. Soc. Appl. Res. Mem. Cogn..

[27] Piccardi L., Iaria G., Ricci M., Bianchini F., Zompanti L., Guariglia C. (2008). Walking in the Corsi test: Which type of memory do you need?. Neurosci. Lett..

[28] Juan-Espinosa M., Abad F.J., Colom R., Fernandez-Truchaud M. (2000). Individual differences in large-spaces orientation: G and beyond?. Personal. Individ. Differ..

[29] Giancola M., Verde P., Cacciapuoti L., Angelino G., Piccardi L., Bocchi A., Palmiero M., Nori R. (2021). Do Advanced Spatial Strategies Depend on the Number of Flight Hours? The Case of Military Pilots. Brain Sci..

[30] Verde P., Angelino G., Piccolo F., Carrozzo P., Piccardi L., Nori R. (2019). Engineers’ abilities influence spatial perspective changing. Internat. J. Eng. Ed..

[31] Boccia M., Bonavita A., Diana S., Di Vita A., Ciurli M.P., Guariglia C. (2019). Topographical Disorientation: Clinical and Theoretical Significance of Long-Lasting Improvements Following Imagery-Based Training. Front. Hum. Neurosci..

[32] Farrell M.J. (1996). Topographical disorientation. Neurocase.

[33] Burgess N. (2006). Spatial memory: How egocentric and allocentric combine. Trends Cogn. Sci..

[34] Burgess N. (2008). Spatial cognition and the brain. Ann. N. Y. Acad. Sci..

[35] Arleo A., Rondi-Reig L. (2007). Multimodal sensory integration andconcurrent navigation strategies for spatial cognition in real and artificialorganisms. J. Integr. Neurosci..

[36] Burkova V.N., Butovskaya M.L., Randall A.K., Fedenok J.N., Ahmadi K., Alghraibeh A.M., Allami F.B.M., Alpaslan F.S., Al-Zu’bi M.A.A., Al-Mseidin K.I.M. (2022). Factors associated with highest symptoms of anxiety during COVID-19: A cross-cultural study of 23 countries. Front. Psychol..

[37] Bowles D.C., McKone E., Dawel A., Duchaine B., Palermo R., Schmalzl L., Rivolta D., Wilson C.E., Yovel G. (2009). Diagnosing prosopagnosia: Effects of ageing, sex, and participant-stimulus ethnic match on the Cambridge Face Memory Test and the Cambridge Face Perception Test. Cogn. Neuropsychol..

[38] Kennerknecht I., Grueter T., Welling B., Wentzek S., Horst J., Ewards S., Grueter M. (2006). First report of prevalence of non-syndromic heredity prosopagnosia (HPA). Am. J. Med. Genet. Part A.

